# Supplementary material for: Inferring the Clonal Structure of Viral Populations from Time Series Sequencing
Source: PLoS Comput Biol. 2015 Nov 16;11(11):e1004344. doi: 10.1371/journal.pcbi.1004344 (PMC4646700; doi:10.1371/journal.pcbi.1004344)
Supplement: S5 Data — (PDF) [file pcbi.1004344.s005.pdf]

Horse	Day 2	Day 3	Day 4	Day 5	Day 6
**2761**	2.35E+06	1.07E+05	3.20E+04	5.49E+03	5.77E+03
**6652**	Day 2	Day 3	Day 5		
	6.49E+03	3.29E+05	2.05E+04		
**6692**	Day 3	Day 4	Day 5	Day 6	
	6.43E+05	8.65E+04	4.49E+04	4.24E+03	
**1420**	Day 3	Day 5	Day 6		
	4.73E+03	1.13E+04	4.96E+03		
